# Wood volatiles as attractants of the confused flour beetle, *Tribolium confusum* (Coleoptera: Tenebrionidae)

**DOI:** 10.1038/s41598-019-48073-9

**Published:** 2019-08-08

**Authors:** Masatoshi Hori, Yoshimi Aoki, Kazutaka Shinoda, Mitsuo Chiba, Rikiya Sasaki

**Affiliations:** 1 Graduate School of Agricultural Science, Tohoku University, Sendai, Miyagi 980-8572 Japan; 2 Insect Pheromone & Traps Division, Fuji Flavor Co., Ltd., Hamura, Tokyo 205-8503 Japan; 3 Present Address: Pest Control Engineering Department, Seibu Kasei Co., Ltd., Shimonoseki, Yamaguchi 750-0067 Japan

**Keywords:** Chemical ecology, Entomology

## Abstract

Confused flour beetles are serious pests of stored grain products, and therefore, it is important to efficiently monitor and control their populations. Aggregation pheromones are commercially used for monitoring this beetle but their efficacy has been questioned and they may be inadequate for practical use. Food attractants as well as pheromones are commonly used for monitoring stored-product insects. However, food attractants may not be effective in the case of food handling facilities, which are already filled with food odours. The ancestors of flour beetles may have been associated with dead or decomposing woody vegetation, so we investigated the attractiveness of several wood odours to beetles using a pitfall olfactometer. The beetles were strongly attracted to all wood odours tested: *Castanea crenata*, *Magnolia obovata*, *Paulownia tomentosa*, *Prunus jamasakura*, and *Zelkova serrata*. The attractiveness of these wood odours was also stronger than that of the odours of the usual food of these beetles. Supercritical CO_2_ extracts of these species of wood were also attractive to the beetles. The *Z*. *serrata* extract was the most attractive among these extracts, and was further analysed by gas chromatography mass spectrometry. One major compound, (−)-mellein, was detected in the extract. Synthetic (±)-mellein attracted the beetles.

## Introduction

The large-scale use of insecticides for controlling stored-product insects is not desirable in food processing or storage facilities because of the risk of contaminating food products. Therefore, it is important to monitor these pests so that effective management strategies can be employed to maintain good levels of hygiene based on data pertaining to the pests collected through effective monitoring traps. Although repellents have been studied for several stored-product insects such as *Lasioderma serricorne* (Fabricius)^1–3^, *Callosobruchus chinensis* (L.)^4^, *Tribolium confusum* Jacquelin du Val, and *Sitophilus zeamais* Motschulsky^5^, their effective application methods in food facilities are limited.

The confused flour beetle, *T*. *confusum* (Coleoptera: Tenebrionidae) is a worldwide serious insect pest of stored grains and their products, similar to the well-known red flour beetle, *T*. *castaneum* (Herbst). Aggregation pheromones are commonly used as an attractant in the monitoring traps of the flour beetles in food facilities^6–11^. However, it has been found that the attractiveness of the aggregation pheromones currently used is limited^12,13^, reducing their efficacy. Therefore, it is important to develop more effective alternative attractants for these beetles. Food attractants are also commonly used in the monitoring traps for stored insect pests^14–16^, and there are many reports on the attractiveness of host-food odours to insects^17–24^. In the flour beetles also, food volatiles have been well studied as attractants for traps^6,20,21,25^; however, their efficacy is limited in food facilities^26^ owing to the high occurrence of food odours already present.

The stored-product pests of the family Tenebrionidae belong, with few exceptions, to the subfamily Ulominae^27^. Most members of this subfamily live under bark or in rotting wood as semipredators and scavengers^27^. The various species of *Tribolium*, which belong to the Ulominae, are also to be found under bark^28^. It is considered that *Tribolium* have originally occurred primarily in rotting logs and under tree bark feeding on plant and animal detritus, and on insect eggs and pupae^29,30^. Therefore, it is reasonable to assume that *T*. *confusum* and *T*. *castaneum* may be attracted to odours from these woody habitats. If these odours are effective at attracting these beetles, then they should also be effective in food facilities as wood odours significantly differ from food odours. In this study, we investigated the attractiveness of several wood odours to *T*. *confusum* and found them to be effective attractants. Our findings suggest that odours of the original habitats or foods of stored-product pests are the promising resources for screening attractants for them.

## Materials and Methods

### Insects

Adults of the confused flour beetle, *Tribolium confusum* Jacquelin du Val, maintained in our laboratory were used for the tests. They were reared on wheat flour (‘Yukichikara’; Sugawara Seifun Seimen Kojo, Iwate, Japan) containing 5% dry brewer’s yeast (Ebios^®^; Asahi Food & Healthcare Co., Tokyo, Japan), and maintained at 25 ± 1 °C and ca. 70% relative humidity under a 16L:8D photoperiod.

### Stored food products

We investigated the attractiveness of the odours of five stored food products that are suitable food for *T*. *confusum*: wheat flour (‘Yukichikara’; Sugawara Seifun Seimen Kojo, Iwate, Japan), polished rice (‘Sasanishiki’, milling of rice grains to 90–92% of weight), unpolished rice (‘Hitomebore’), corn flour (Nippon Flour Mills Co., Tokyo, Japan), and soybean flour (Matsuda Seifun Co., Miyagi, Japan). The polished rice and unpolished rice were pulverised using a mill (Sibata Personal Mill, SCM-40A, Sibata Scientific Technology Ltd., Saitama, Japan) in preparation for the tests.

### Wood

We used five species of wood, *Castanea crenata*, *Magnolia obovata*, *Paulownia tomentosa*, *Prunus jamasakura*, and *Zelkova serrata*, for the tests. All species of timber (1–2 m long) used for the tests were purchased from Kanayama-Chip-Center Co., Ltd., Gifu, Japan. The diameters of the purchased timber were ca. 400, 450, 150, 200, and 150 mm for *C*. *crenata*, *M*. *obovata*, *P*. *tomentosa*, *P*. *jamasakura*, and *Z*. *serrata*, respectively. Each piece of timber was naturally seasoned in the laboratory of Fuji Flavor Co., Ltd. (Kanagawa, Japan) at room temperature (24 ± 3 °C) for about three months after purchase. For attractiveness of wood odours, each piece of timber was divided into wooden blocks (ca. 50 mm long) using an electric saw. Wooden blocks were crushed into chips using a crusher (Wonder Blender^®^, WB-1; Osaka Chemical Co., Ltd., Osaka, Japan). Chips were naturally dried in the laboratory of Tohoku University (Sendai, Japan) at room temperature (24 ± 3 °C) for more than two weeks. Once dry, the chips were pulverised using the mill and were used for the bioassay.

### Supercritical carbon dioxide (CO_2_) extraction of wood

All species of wood were chipped using an electric wood planer (M192, Makita Co., Aichi, Japan). Chips of each type of wood were crushed using a hammer crusher (NH-34, Sansho Industry Co., LTD., Osaka, Japan) and sifted using a 0.4-mm meshed sieve. The powder of each species of wood was extracted using supercritical CO_2_ using extraction apparatus from a plant of Fuji Flavor Co., Ltd. (Tokyo, Japan). Crude extracts were separated into lipid-soluble and water-soluble phases. The volatility and dispersibility of attractants are important in practical use. Lipid-soluble phases usually have more abundant volatile components than water-soluble phases. Therefore, in this study, the water-soluble phases were discarded, and only the lipid-soluble phases were used as extracts. Extraction pressure, extraction temperature, solvent ratio, separation pressure, and separation temperature were 25 MPa, 15 °C, 20:1, 4 MPa, and 20 °C, respectively. The extracts containing solids were centrifuged at 8000 g for 10 min to remove them. The weight of the wood used for the extractions and the weight of each extract obtained were as follows: *C*. *crenata* (wood powder: 27.62 kg; extract: 30 g), *M*. *obovata* (17.92 kg; 140 g), *P*. *tomentosa* (30.42 kg; 280 g), *P*. *jamasakura* (29.14 kg; 50 g), and *Z*. *serrata* (28.47 kg; 20 g).

### Chemical analysis of supercritical CO_2_ extract of *Z*. *serrata*

The supercritical CO_2_ extracts of *Z*. *serrata* were analysed by GC-MS (Shimadzu GCMS-QP2010 Ultra, equipped with a DB-5MS column, 30 m × 0.25 mm i.d., 0.25 μm film thickness, J&W, Santa Clara, CA, USA). The extract was diluted to 5% (w/w) in hexane and filtered through a Minisart^®^ RC15 filter (0.45 μm pore size; Sartorius Stedim Biotech, Göttingen, Germany). The filtrate (1 μl) was injected into the GC/MS. Helium was used as the carrier gas at a column head pressure of 100 kPa. The GC was set for split injection (split ratio, 100:1). The temperature program of the column oven was as follows: initial temperature 60 °C, 3 °C/min to 195 °C. The injector, detector, and interface temperatures were 220 °C, 200 °C, and 240 °C, respectively. Mass spectral data were analysed using Shimadzu GCMS Solution with the Wiley Registry (9^th^ ed.), National Institute of Standards and Technology (NIST05), and Flavor and Fragrance Natural and Synthetic Compounds (FFNSC ver. 1.3) mass spectral databases. Major compounds of the extract were identified by comparing GC retention times and mass spectra with those of the authentic (±)-mellein synthesised in Fuji Flavor Co., Ltd. The concentration of mellein in the *Z*. *serrata* extract was calculated from the calibration curve for the authentic mellein.

In order to identify the enantiomer of mellein in the *Z*. *serrata* extract, (−)-mellein was purchased from Funakoshi Co., Ltd (Tokyo, Japan). Mellein in the extract, (±)-mellein and (−)-mellein were analysed by GC-MS (Shimadzu GCMS-QP2010 Ultra, equipped with an InterCap CHIRAMIX column, 30 m × 0.25 mm i.d., 0.25 μm film thickness, GL Sciences Inc., Tokyo, Japan). Helium was used as the carrier gas at a column head pressure of 100 kPa. The GC was set for split injection (split ratio 100:1). The temperature program of the column oven was as follows: isotherm for 180 min at 130 °C, 10 °C/min increase to 150 °C, and isotherm for 20 min at 150 °C. The injector, detector, and interface temperatures were 230 °C, 250 °C, and 180 °C, respectively. Retention time of the peak of mellein in the extract was compared with those of ( ± )-mellein and (−)-mellein.

### Bioassay

Behavioural responses of *T*. *confusum* to the wood odours were investigated with the pitfall-trap olfactometer (Supplementary Fig. 1). The olfactometer comprised a glass Petri dish (246 mm dia.) with four round holes (30 mm) with four plastic containers (60 × 60 × 100 mm) set under the holes. The containers acted as traps for the beetles. Two out of the four containers were used as treatment traps and the others were used as control traps. The treatment traps and the control traps were arranged alternately in all the experiments. Each hole was covered with a piece of polyethylene mesh (opening of mesh: 5 mm). A sheet of filter paper with four round holes (30 mm) was stuck on the Petri dish so that the holes overlapped with those of the Petri dish. The beetles walking on the filter paper were attracted to the odours and fell into the traps through the meshes of the holes. Fifty beetles of 3- to 4-week-old unsexed adults that were starved for 24 h were released on to the centre of the filter paper. All bioassays were conducted at 25 ± 1 °C and approximately 70% relative humidity under dark conditions to remove any potential influence of light on behavioural responses. The total number of beetles in the two treatment traps was compared with the two control traps 3 h after the start of the assay. In the preliminary tests, approximately equal number of beetles were trapped in each of the four traps when all traps were empty. Each comparison was replicated 10 times; except for the assays using mellein, in which eight replications were conducted for each dose. The glass Petri dish and the traps of the olfactometer were washed with soapy water (LIQUINOX^®^; Alconox, Inc., White Plains, NY, USA), and the sheet of filter paper and the pieces of mesh were replaced after every replication. The positions of the treatment and control traps were alternated after half of the replications had been completed.

The total number of beetles in the traps of the treatment and control groups were analysed using a Wilcoxon matched-pairs signed-ranks test. The attractiveness of each sample to the beetles was estimated by a response index according to the formula:

$$RI=100\times (nt-\,nc)/tot$$

where *nt*, *nc*, and *tot* represent the total number of beetles in the treatment and control traps, and the total number of beetles released in the olfactometer, respectively.

In the tests of attractiveness of wood odours, test wood or food material (2 g) was placed in each of two treatment traps; the two control traps were empty. In the tests of attractiveness of extracts and mellein, a disk (12 mm dia., 3 mm thickness, material: polyethylene/ethylene-vinyl acetate copolymerisation fibre) impregnated with 100 μl of the acetone solution of each sample (doses of extract: 0.1, 1, or 10 mg/disk; doses of mellein: 0.05, 0.1, 1, and 10 mg/disk) was placed in each treatment trap, whereas a disk impregnated with only 100 μl acetone was placed in each control trap. Both treatment and control disks were allowed to dry to remove the acetone before they were placed in the traps. In the choice test between the *Z*. *serrata* extract and wheat flour odour, wheat flour (2 g) was placed in each control trap and the control disk impregnated with only 100 μl of acetone was placed on the wheat flour, whereas only the treatment disk impregnated with 100 μl of the acetone solution of *Z*. *serrata* extract (extract dose: 1 mg/disk) was placed in each treatment trap. In attractiveness of mellein, (±)-mellein synthesised by Fuji Flavor Co., Ltd. was used because of the cost. The cost of either asymmetrically synthesising or purchasing (−)-mellein was too high for use in the bioassay.

## Results

### Attractiveness of wood odours

**Figure 1:**
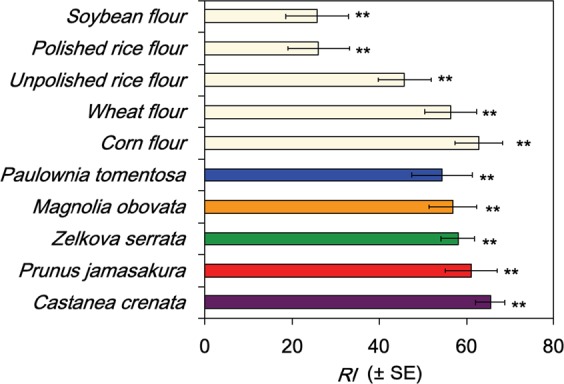
Attractiveness of the wood and food odours to *Tribolium confusum* in pitfall-trap olfactometer tests. Test wood or food material (2 g) was placed in each of two treatment traps; the two control traps were empty. Fifty adult beetles starved for 24 h were tested for each replication. The total number of beetles in the two treatment traps was compared with the two control traps 3 h after the start of the assay. *RI* = 100 × (*nt* − *nc*)/*tot*, where *nt*, *nc*, and *tot* represent the total number of beetles in the treatment and control traps, and the total number of beetles released in the olfactometer, respectively. ** indicates significant differences between the treatment and control traps (Wilcoxon matched-pairs signed-ranks test: *P* < 0.01, *n* = 10).

We investigated the attractiveness of odours from five species of wood: *Castanea crenata*, *Magnolia obovata*, *Paulownia tomentosa*, *Prunus jamasakura*, and *Zelkova serrata*, to *T*. *confusum*, using a pitfall-trap olfactometer. In addition, the attractiveness of these odours was compared to that of odours of suitable foods for the beetles, i.e. polished rice, unpolished rice, wheat flour, corn flour, and soybean flour. All five wood odours were similarly attractive to the beetles showing response index (RI) values of ca. 55–65, which were similar levels to those of unpolished rice, wheat, and corn flours, and were considerably higher than those of soybean and polished rice flours (Fig. 1). The odour of *C*. *crenata* showed the highest RI value, i.e. 65.4. This value was similar to that of the corn flour odour (RI value: 62.8), which showed the highest attractiveness among food odours.

### Attractiveness of wood extracts

**Figure 2:**
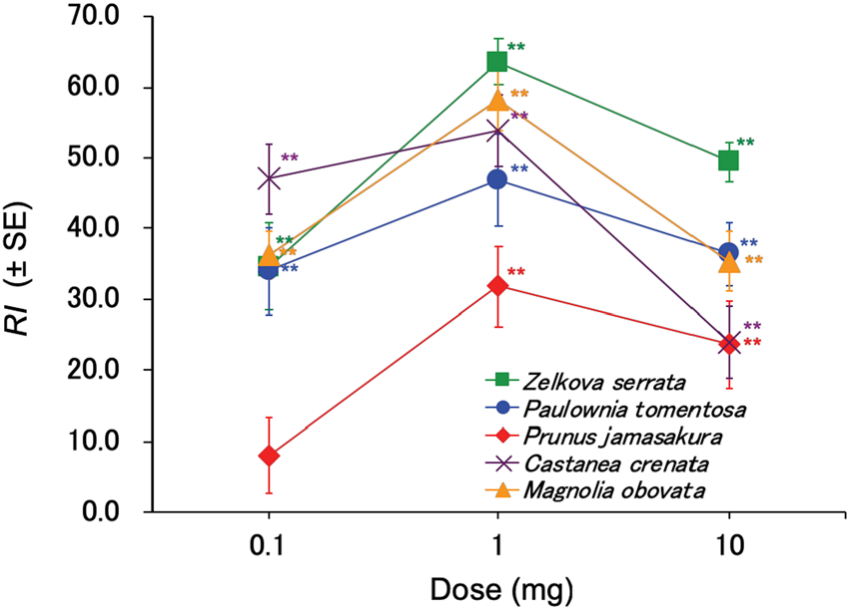
Attractiveness of the supercritical CO_2_ extracts of woods to *Tribolium confusum* in pitfall-trap olfactometer tests. A disk impregnated with 100 μl of acetone solution of each sample was placed in each of the two treatment traps, whereas a disk impregnated with only 100 μl acetone was placed in each of the two control traps (50 adult beetles starved for 24 h per replication). The total number of beetles in the two treatment traps was compared with the two control traps 3 h after the start of the assay. *RI* = 100 × (*nt* − *nc*)/*tot*, where *nt*, *nc*, and *tot* represent the total number of beetles in the treatment and control traps, and the total number of beetles released in the olfactometer, respectively. ** indicates significant differences between the treatment and control traps (Wilcoxon matched-pairs signed-ranks test: *P* < 0.01, *n* = 10).

In the tests for attractiveness of wood odours, the odours of all five species of wood tested were highly attractive to *T*. *confusum*; therefore, we investigated the attractiveness of supercritical carbon dioxide (CO_2_) extracts of these five species of wood using the pitfall-trap olfactometer. All extracts tested showed the highest attractiveness at a dose of 1 mg among the doses tested. The extract of *Z*. *serrata* exhibited the highest activity with a RI value of 63.6 at a dose of 1 mg (Fig. 2). The attractiveness of the extract of *P*. *jamasakura*, of which the wood odour showed the highest level of activity next to that of *C*. *crenata* (Fig. 1), was the lowest performing among the extracts tested. The extract of *C*. *crenata* showed the highest attractiveness among the extracts tested at a dose of 0.1 mg and relatively high attractiveness at a dose of 1 mg, whereas it showed the lowest attractiveness, similar to that of *P*. *jamasakura* at a dose of 10 mg.

### Choice test between the *Z*. *serrata* extract and wheat flour odour

In the tests for attractiveness of wood extracts, the *Z*. *serrata* extract showed the highest attractiveness at doses of 1 and 10 mg among the extracts tested. Hence, we allowed *T*. *confusum* to choose between the *Z*. *serrata* extract (1 mg) and wheat flour (2 g) odours using the pitfall-trap olfactometer. The beetles chose the *Z*. *serrata* extract showing a RI value of 35.2 significantly more often (*P* < 0.01, Wilcoxon matched-pairs signed-ranks test, *n* = 10).

### Isolation and identification of semiochemicals from *Z*. *serrata* extract

**Figure 3:**
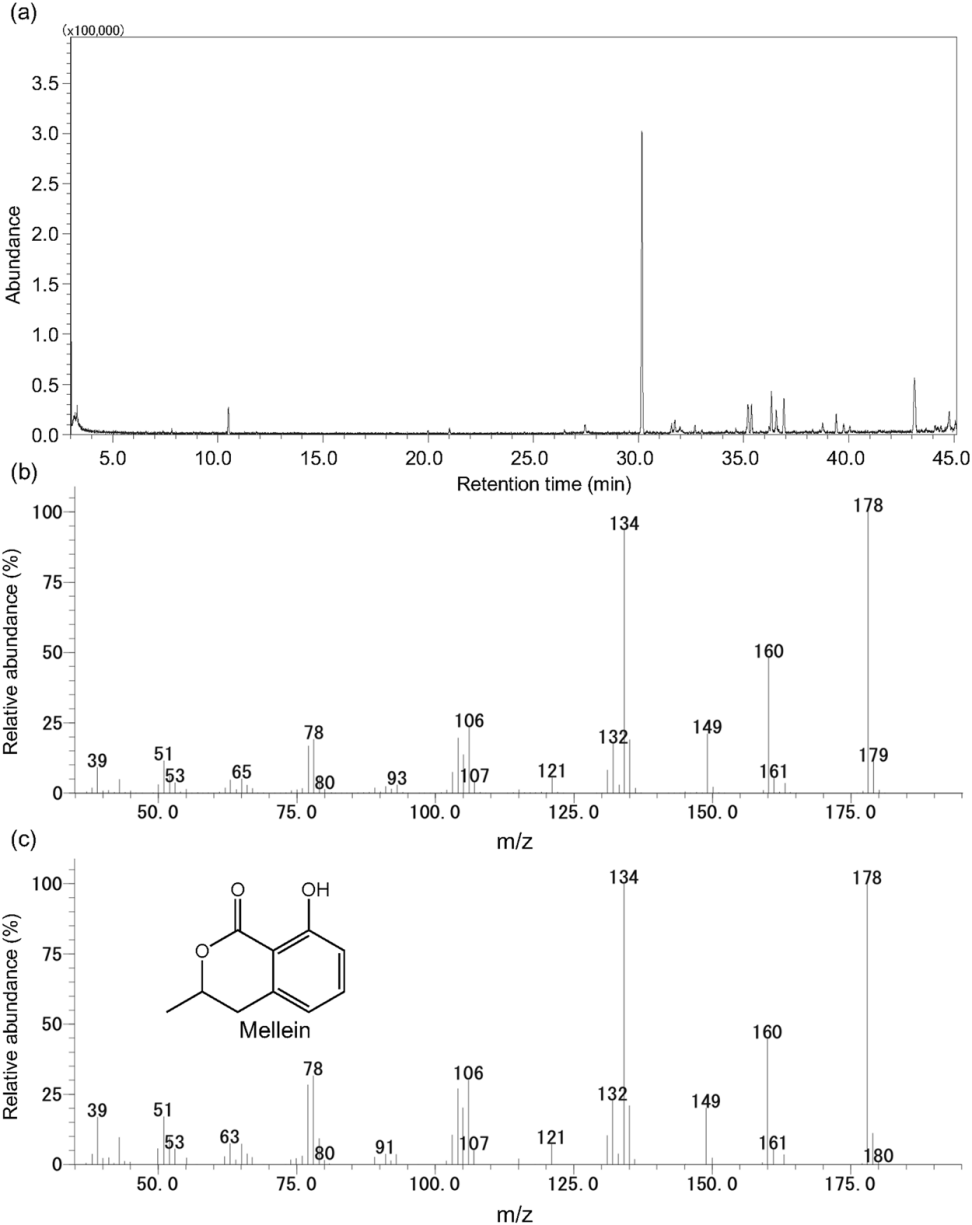
Gas chromatography/mass spectrometry analysis of supercritical CO_2_ extracts of *Zelkova serrata*. (**a**) Total ion chromatogram of the *Z*. *serrata* extract. (**b**) Mass spectrum of the peak detected at 30.2 min. (**c**) Mass spectrum of the authentic sample of 3,4-dihydro-8-hydroxy-3-methyl-1*H*-2-benzopyran-1-one (mellein).

We analysed the volatile compounds with low polarity contained in the *Z*. *serrata* extract by coupled gas chromatography/mass spectrometry (GC/MS). In the total ion chromatogram of the *Z*. *serrata* extract, one major peak was detected at the retention time of 30.2 min (Fig. 3a). This compound was identified as 3,4-dihydro-8-hydroxy-3-methyl-1*H*-2-benzopyran-1-one (mellein) by comparing the gas chromatograph retention time and the mass spectra with those of a known sample of mellein (Fig. 3b,c). It was quantitatively analysed by comparing the peak area of total ion chromatogram of mellein contained in the *Z*. *serrata* extract with that of a known sample of mellein, which showed that the mellein concentration in the extract was approximately 0.5%.

To identify the enantiomer of mellein in the *Z*. *serrata* extract, we compared the chiral GC/MS peak of mellein in the extract to those of (±)-mellein and (−)-mellein. Mellein in the extract and (−)-mellein showed one peak at a retention time of 191.0 min, whereas (±)-mellein showed two peaks at retention times of 190.3 min and 191.0 min. Therefore, the mellein in the extract was identified as the (−) enantiomer.

### Attractiveness of mellein

**Figure 4:**
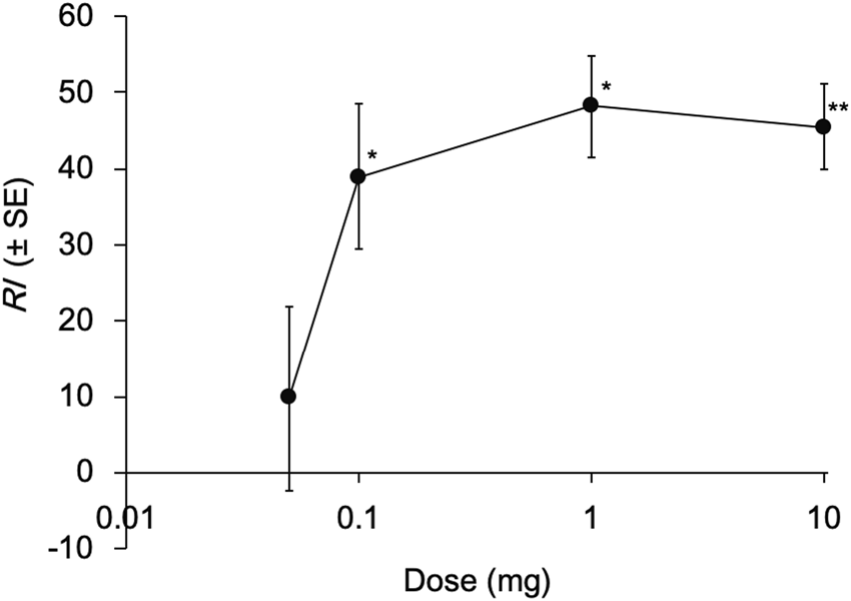
Attractiveness of (±)-mellein to *Tribolium confusum* in the pitfall-trap olfactometer tests. A disk impregnated with 100 μl of acetone solution of (±)-mellein was placed in each of the two treatment traps, whereas a disk impregnated with only 100 μl acetone was placed in each of the two control traps (50 beetles starved for 24 h per replication). The total number of beetles in the two treatment traps was compared with the two control traps 3 h after the start of the assay. *RI* = 100 × (*nt* − *nc*)/*tot*, where *nt*, *nc*, and *tot* represent the total number of beetles in the treatment and control traps, and the total number of beetles released in the olfactometer, respectively. ** indicates significant differences between the treatment and control traps (Wilcoxon matched-pairs signed-ranks test: *P* < 0.01, *n* = 8).

(±)-Mellein significantly attracted *T*. *confusum* at doses of 0.1–10 mg showing a dose-response relationship (Fig. 4). RI values were ca. 40, 50, and 50 at doses of 0.1, 1, and 10 mg, respectively. Although the *Z*. *serrata* extract significantly attracted the beetles at a dose of 0.1 mg, which contains 0.0005 mg of mellein (Fig. 3), 0.05 mg of mellein was not attractive to the beetles (Fig. 4).

## Discussion

The woods tested in this study belong to five different families representing four diverse orders of plants: *C*. *crenata*, *M*. *obovata*, *P*. *tomentosa*, *P*. *jamasakura*, and *Z*. *serrata* belong to Fagaceae (Fagales), Magnoliaceae (Magnoliales), Paulowniaceae (Lamiales), Rosaceae (Rosales), and Ulmaceae (Rosales), respectively. However, all wood odours were attractive to *T*. *confusum* and were similar in attractiveness to the odours of suitable foods for the beetles. Therefore, the beetles were attracted to wood odours from a variety of families and orders, which are not necessarily closely related. It is probable that *T*. *confusum* would be attracted to wide range of wood odours. It is considered that *Tribolium* originally fed on plant and animal detritus, and on insect eggs and pupae in rotting logs and under tree bark^29,30^. The positive chemotaxis of *T*. *confusum* to odours from various species of wood observed in the present study may be related to the original habitat of this beetle. It is believed that food storage by ancient people began over 10,000 years ago^31^, offering a more stable habitat for insects, more sheltered from environmental changes and with a more abundant, steady supply of food. Certain species of *Tribolium* adapted to this environment^32^, losing their close association with rotting logs and bark leading to species with no association with these habitats, such as *T*. *confusum*^33^. The response of these beetles to woody odours observed in the present study suggests that olfactory recognition of the odours of their original habitat is likely to have been maintained genetically.

In this study, we investigated the olfactory responses of the beetles to odours of naturally seasoned timber using a pitfall-trap olfactometer. Therefore, the role of these findings in understanding chemotaxis in the natural environment is still unclear. In some *Dendroctonus* beetles (bark beetles), it is believed that pioneers can locate their coniferous hosts by attraction to volatiles and followers can quickly locate the hosts by using pheromones^34^. Further research with field trapping using aggregation pheromone and the volatiles of intact and decomposing trees and barks would help to clarify the specific chemotaxis in *Tribolium* beetles related to innate sexual, gregarious, or host-seeking behaviours.

Males of *T*. *confusum* produce an aggregation pheromone^35,36^; however, a previous study using the same pitfall-trap olfactometer as the present study found that the odour of conspecific males or females at high density (50 conspecifics/trap), without food odour, repelled both sexes, and that the odour at low density (5 or 10 conspecifics/trap) neither repelled nor attracted them^37^. Additionally, a high density of conspecifics did not suppress the attractiveness of wheat-flour odour to the beetles^37^. Therefore, we considered that the olfactory responses of the beetles to food-related volatiles observed in the present study were not affected by conspecific odours. For baited traps to be effective, it is required that the odour of the beetles initially trapped does not negatively affect the attractiveness of the trap to others. This study suggests that this is not the case for the attractiveness of wood odours.

Aggregation pheromones are commonly used in traps to monitor population of *T*. *confusum*, as well as *T*. *castaneum* in food handling facilities^6–11^ to aid in decisions to control them, but their efficacy has been questioned^12,13^. Attractants screened from the volatile compounds of the beetle’s food have also commonly been used for traps^17–20,23^. The efficacy of this method is, however, questionable, as food facilities are already filled with food odours, so it is difficult for the insects to recognise food attractants, being a useful pheromone synergist, volatilised from the traps^26^. If the odours from the original foods or habitats of the beetles differ from those of the stored foods, then the insects may easily discriminate between them. In the choice test between the *Z*. *serrata* extract and wheat flour odour, *T*. *confusum* significantly chose the *Z*. *serrata* extract compared with the wheat flour odour. Therefore, it is likely that volatile compounds from materials from the original habitat of stored-product insects can be useful as attractants.

It has been reported that mellein and its analogues have been isolated from trees^38^ and fungus in their bark^39–41^. Therefore, it is thought that the mellein identified in this study is from the *Z*. *serrata* trees itself and/or the fungus in the trees. In the current study, mellein was also detected as a minor compound in the *M*. *obovata* extract. Therefore, mellein may be one of the odours that attract *T*. *confusum* to the bark of trees. It is well known that mellein is produced by *Aspergillus* fungi, such as *A*. *ochraceus* and *A*. *flavus*^42–44^, which are common in soil, stored grains, and rotting wood^39,45–49^. The attraction of mellein might have played a role in the connection between original and current habitats.

The attractiveness of mellein was lower than that of the *Z*. *serrata* extract. In addition, the mellein concentration contained in the extract was approximately 0.5%. In attractants used for traps, high diffusivity is required for the trap to be effective over long distances. We analysed all wood extracts by GC-MS after being dissolved in hexane and filtered to determine the volatility of their components, in order to identify highly volatile components with low polarity contained in the extracts. The results suggest that other attractants or synergists of mellein that are low-volatile components of high polarity, are likely to be contained in the *Z*. *serrata* extract. It is possible that some other attractants or synergists in addition to mellein occur in the lipid aqueous fractions not studied here.

In the current study, we used (±)-mellein in the bioassay although only the (−) enantiomer was contained in the *Z*. *serrata* extract. It has been reported that some insect species can discriminate between enantiomers of certain volatile compounds^50–55^. It is thought that the lower activity of (±)-mellein compared to that of the *Z*. *serrata* extract may be due to interference with the attraction by the (+) enantiomer which is not contained in the extract. Further study is needed on the differences in activity between each enantiomer and *T*. *confusum*.

To the best of our knowledge, this is the first study to show that the odours from the habitats or foods of stored-product insects, prior to their adaptation to human foods, may be useful as attractants in the traps used to monitor them. Beside *T*. *confusum*, many species of stored-product insects may be attracted to the odours of their original habitats or foods. This study provides novel ideas on the resources for screening attractants for stored-product insects.

## Conclusions

*T*. *confusum* were attracted to wood-based odours, which are believed to correspond with their original habitats or foods, even more than to their usual food. This suggests that these wood-based odours would be ideal for use in monitoring traps and should be considered for future efforts to control stored-product pests.

## Supplementary information

Supplementary Figure 1

## Data Availability

The datasets are available from the corresponding author on reasonable request.
