# Supplementary material for: Wood volatiles as attractants of the confused flour beetle, Tribolium confusum (Coleoptera: Tenebrionidae)
Source: Sci Rep. 2019 Aug 8;9:11544. doi: 10.1038/s41598-019-48073-9 (PMC6687883; doi:10.1038/s41598-019-48073-9)
Supplement: Supplementary file 1 — Supplementary Figure 1 [file 41598_2019_48073_MOESM1_ESM.pdf]

# **Wood volatiles as attractants of the confused flour beetle, *Tribolium confusum* (Coleoptera: Tenebrionidae)**

Masatoshi Hori[1*], Yoshimi Aoki[1], Kazutaka Shinoda[2], Rikiya Sasaki[2] & Mitsuo Chiba[2]

[1]Graduate School of Agricultural Science, Tohoku University, Sendai, Miyagi 980-0845, Japan

[2]Insect Pheromone & Traps Division, Fuji Flavor Co., Ltd., Hamura, Tokyo 205-8503, Japan

[*]Correspondence: hori@tohoku.ac.jp

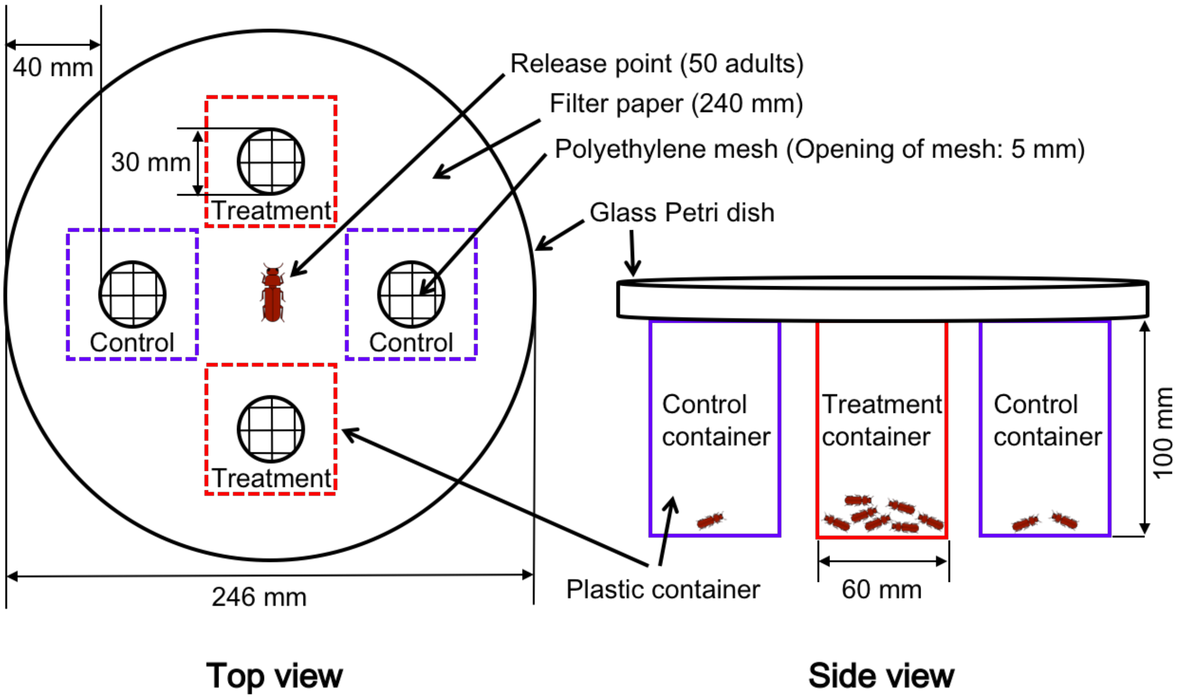


**Supplementary Figure 1.** Pitfall-trap olfactometer used for the bioassays.
